# Analysis of Predicted Host–Parasite Interactomes Reveals Commonalities and Specificities Related to Parasitic Lifestyle and Tissues Tropism

**DOI:** 10.3389/fimmu.2019.00212

**Published:** 2019-02-13

**Authors:** Yesid Cuesta-Astroz, Alberto Santos, Guilherme Oliveira, Lars J. Jensen

**Affiliations:** ^1^Instituto René Rachou, Fundação Oswaldo Cruz - FIOCRUZ, Belo Horizonte, Brazil; ^2^Novo Nordisk Foundation Center for Protein Research, Faculty of Health and Medical Sciences, University of Copenhagen, Copenhagen, Denmark; ^3^Environmental Genomics, Instituto Tecnológico Vale, Belém, Brazil

**Keywords:** computational biology, systems biology, biological networks, parasitology, schistosomiasis, host–parasite interactions

## Abstract

The study of molecular host–parasite interactions is essential to understand parasitic infection and adaptation within the host system. As well, prevention and treatment of infectious diseases require a clear understanding of the molecular crosstalk between parasites and their hosts. Yet, large-scale experimental identification of host–parasite molecular interactions remains challenging, and the use of computational predictions becomes then necessary. Here, we propose a computational integrative approach to predict host—parasite protein—protein interaction (PPI) networks resulting from the human infection by 15 different eukaryotic parasites. We used an orthology-based approach to transfer high-confidence intraspecies interactions obtained from the STRING database to the corresponding interspecies homolog protein pairs in the host–parasite system. Our approach uses either the parasites predicted secretome and membrane proteins, or only the secretome, depending on whether they are uni- or multi-cellular, respectively, to reduce the number of false predictions. Moreover, the host proteome is filtered for proteins expressed in selected cellular localizations and tissues supporting the parasite growth. We evaluated the inferred interactions by analyzing the enriched biological processes and pathways in the predicted networks and their association with known parasitic invasion and evasion mechanisms. The resulting PPI networks were compared across parasites to identify common mechanisms that may define a global pathogenic hallmark. We also provided a study case focusing on a closer examination of the human–*S. mansoni* predicted interactome, detecting central proteins that have relevant roles in the human–*S. mansoni* network, and identifying tissue-specific interactions with key roles in the life cycle of the parasite. The predicted PPI networks can be visualized and downloaded at http://orthohpi.jensenlab.org.

## Introduction

Parasites are responsible for many diseases that result in millions of deaths each year. For instance, the World Health Organization published data in 2016 estimating that *Plasmodium falciparum* alone was responsible for around 214 million malaria cases, and 438,000 deaths worldwide (1). As well, around 7 million people worldwide were reported to be infected with *Trypanosoma cruzi*, which causes Chagas disease that results in life-long morbidity and disability and more than 7,000 deaths per year (1). Another highly prevalent disease, Leishmaniasis accounts for 20 to 30 thousand deaths a year and is caused by protozoan parasites of the *Leishmania* genus (1). Similarly, Schistosomiasis, a neglected parasitic disease of high relevance in this work, is mainly caused by five species of the genus *Schistosoma*. The disease has an estimated prevalence of 200 million cases worldwide (2). The available treatment for schistosomiasis is limited, and the development of resistance is a concern. Thus, there is an urgent need to develop novel drugs or vaccines.

The development of vaccines or treatments has been impeded by the lack of understanding of the parasites infection and survival mechanisms. Typically, parasites have complex life cycles with several morphological stages and infect distinct host cell types and tissues. For that, parasites display a resourceful capacity to live in different environmental conditions (intra and extracellular parasites) and also resist the immunological response of hosts (3). For example, extracellular parasites remodel tissues to migrate and evade the immune system (4). Similarly, intracellular parasites shape cellular processes and remodel host cells to adjust their niche during infection (5). The manipulation of these processes and pathways happens through molecular interactions that parasites use to their advantage.

The study of molecular host–parasite interactions is essential to understand parasitic infection, local adaptation within the host, and pathogenesis. These complex interactions can be described as a network (6). Pathogens affect their hosts partly by interacting with host proteins, which defines a molecular interplay between the parasite survival mechanisms and the host's defense and metabolic systems (7). Understanding this molecular crosstalk can provide insights into specific interactions that could be targeted to avoid the pathological outcomes resulting from the parasitism (8).

Intra-species protein–protein interactions (PPIs) have been studied in depth and there exist large datasets containing experimentally or computationally predicted interactions (9, 10). However, the number of available datasets providing host–pathogen PPIs is limited and challenged by the intrinsic difficulties of analyzing simultaneously both host and pathogen systems in high-throughput experiments (11). Thus, host–pathogen PPIs have mainly been predicted computationally using distinct strategies such as approaches based on sequence (8, 12–15), structure (16, 17), and gene expression (18). Homology-based prediction is one of the most common approaches to predict host–pathogen PPIs. These approaches have been extensively used to infer intra-species interactions (10, 14, 19–22) as well as host–pathogen PPIs (13, 15, 17, 23) based on the assumption that interactions between proteins in one species can be transferred to homolog proteins in another species (interologs).

In this work, we have followed a similar prediction strategy to identify common and specific mechanisms of parasitic infection and survival across 15 human parasites, namely *Trypanosoma brucei, Trypanosoma cruzi, Trichinella spiralis, Schistosoma mansoni, Giardia lamblia, Plasmodium falciparum, Plasmodium vivax, Plasmodium knowlesi, Cryptosporidium hominis, Cryptosporidium parvum, Toxoplasma gondii, Leishmania braziliensis, Leishmania mexicana, Leishmania donovani, and Leishmania infantum*. Our computational prediction approach is based on orthology transfer. However, we have constrained the method by (1) incorporating only high-confidence intra-species interactions, and interactions mined from the scientific literature (10), (2) using fined-grained orthology assignments instead of simple sequence similarity, and (3) including parasite-specific biological context such as lifestyle (uni- or multi-cellular) and tissue infection.

The objective of these constraints was to reduce the number of falsely predicted interactions, increase reliability, and thereby provide a better understanding of the parasites' molecular mechanisms of interaction with the host. The evaluation of the predicted host–parasite PPIs requires repositories of high-throughput experimental data that are not yet existent. However, we present here extended literature references supporting some of the predicted interactions relevant in the host–parasite molecular crosstalk. We also evaluated the predicted PPIs based on the functional relevance by identifying significantly enriched processes annotated in the human proteins predicted to interact with parasites. Finally, we propose that this approach can be applied to any host–pathogen system to predict relevant molecular interactions and define the context in which they unfold.

## Materials and Methods

### Proteome Filtering: Adding Parasite-Specific Biological Context

**Figure 1:**
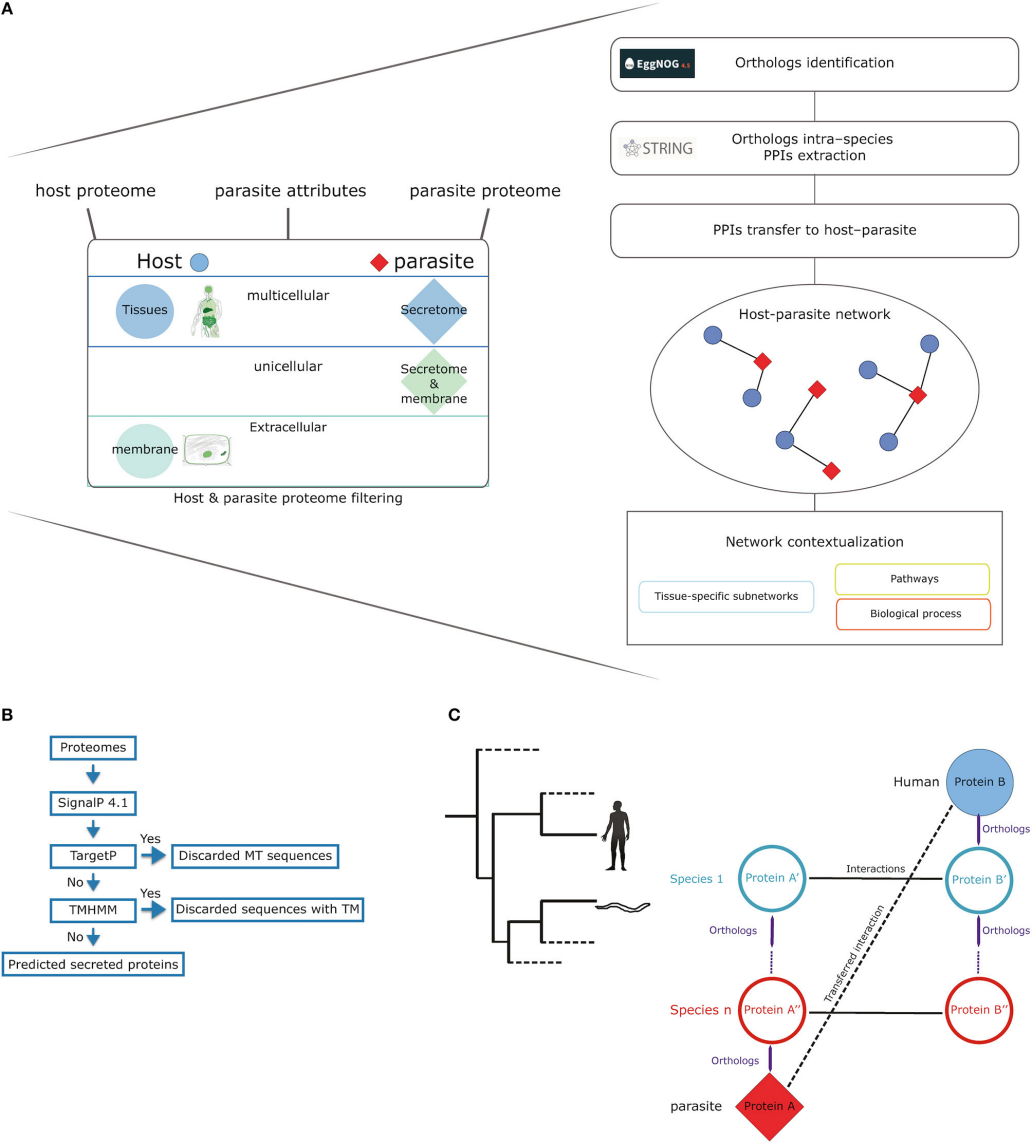
Method workflow. **(A)** The method input consists of the host and parasite proteomes, parasite attributes (uni- or multicellular), and tropism (tissues). Our approach filters the host proteome according to the specified list of tissues using TISSUES database and limits it to only cellular membrane and extracellular proteins extracted from the COMPARTMENTS database. The parasite proteome is filtered to include membrane and secreted proteins (unicellular parasites) or only secreted proteins (multicellular parasites). The next step in the pipeline uses the eggNOG database to identify orthologous proteins for both the filtered proteomes of both the host and the parasite. The workflow continues with the extraction of intra-species protein–protein interactions from STRING database for all the orthologous proteins. These intra-species interactions are then transferred to the host–parasite system as long as the interacting proteins have an orthologous host and parasite proteins. **(B)** Workflow for soluble secretome prediction in parasites (MT, mitochondrial; TM, transmembrane; TMHMM, transmembrane hidden markov model). **(C)** A simple scheme of how the orthology transfer is implemented.

The first step in the prediction pipeline is to filter both the host and parasite proteomes according to the specific characteristics of the studied parasite (Figure 1A). All the human eukaryotic parasites (18 parasites) available in the STRING database were included in our analysis. Firstly, for the interaction to happen, proteins in the parasite need to be secreted or membrane proteins depending on the type of parasite (unicellular or multicellular). When analyzing parasites such as helminths (multicellular and extracellular) we used the soluble secretome. Conversely, in the case of unicellular parasites, we used both the soluble secretome and membrane proteins.

To identify soluble and membrane proteins in parasites, we used different available bioinformatics tools to predict subcellular localization (Figure 1B). SignalP (24) was used to identify classically secreted proteins that were then scanned for the presence of mitochondrial sequences using TargetP (25) and transmembrane helices by the transmembrane identification based on hidden Markov model (TMHMM) method (26). We filtered host proteins to consider only those located on the cellular membrane and extracellular space by using the COMPARTMENTS database, which provides high-confidence information on cellular localization of proteins (confidence score >3) (27).

Additionally, we included context on the specific tissue tropism of the studied parasite by limiting the predictions to host proteins expressed in tissues relevant in the parasite's life cycle, since parasites need to migrate through distinct host tissues to complete their life cycles. Tissue tropism information for each parasite was based in information available on https://www.cdc.gov/parasites/. For this purpose, we used the TISSUES database (28), which provides protein profiles of tissue expression. Protein-tissue associations in this database are also scored in a similar way to the COMPARTMENTS database, which allowed us to use only high-confidence associations (confidence score >3).

The reason for choosing these specific databases is, that they have been designed to work well together. In addition to the wide range of evidence types considered and the consistent scoring schemes, the protein identifiers are synchronized across all of these databases (COMPARTMENTS, TISSUES, eggNOG, and STRING), which enables a smooth integration and a dynamic recovery of orthologs, mapped tissues, and compartments.

### Orthologs Identification and PPIs Transfer

Host–parasite PPIs were predicted using orthology-based transfer. This approach relies on cross-species data integration to predict inter-species protein–protein interactions. Conserved intra-species interactions from multiple organisms, namely interologs, are transferred to the host–parasite system when orthologous proteins exist in these species. For example, an intra-species interaction between protein A and protein B is transferred if the host and the parasite have orthologs to A and B, respectively (Figure 1C).

To obtain intra-species PPIs, we used the STRING database (10). STRING provides PPIs from a variety of sources and evidence types, each with associated confidence scores, which allows us to expand the list of high-confidence PPIs beyond known physical interactions. The interaction file used contains confidence scores for the individual evidence channels (Neighborhood, Gene fusion, Co–occurrence, Co–expression, Experiments, Databases, and Text-mining), which are further subdivided into direct and transferred evidence. The transferred scores come from the orthology transfer performed by the STRING database itself (interologs); we excluded these to only include direct evidences. Consequently, we needed to recalculate the combined score following how scores are combined within the STRING database (Equation 1).

(1) $$\begin{array}{r} \;score_{combined}=1\;-\;\frac{1}{{\left(1-p\right)}^{\left(N-1\right)}}{\;}^{*}\overset{N}{\underset{i=1}{\prod }}\left(1-score_{i}\right), \end{array}$$

where *i* is the different evidence channels (Neighborhood, Gene Fusion, Co–occurrence, Co–expression, Experiments, Databases, and Text-mining) and *p* is the prior probability of two proteins being linked, which is the same value as the one used in the STRING database (*p* = 0.063). The recalculated score was then used to filter for only high-confidence interactions (*score*_*combined*_ > 0.7).

For each of the interactions from STRING, we used the fine-grained orthologs functionality derived from phylogenetic analysis in eggNOG database (29) to identify orthologous proteins in human and in the parasites. We transferred an interaction as long as it involved proteins that had an orthologous protein in the parasite and another one in human among the ones retained in the filtered proteomes. Several metrics were collected to facilitate the analyses of the predicted interactions: maximum confidence score transferred, maximum confidence score transferred from the Experiments channel in STRING, the species from which the interactions were transferred, and the eggNOG non-supervised orthologous groups (NOGs) the proteins belong to.

### Domain-Domain and Linear Motif-Domain Annotations

To know which of the predicted interactions may be physical rather than only functional associations we annotated our interaction predictions with domain–domain interaction predictions from iPfam (30) and 3did (31), and linear motif–domain interactions from ELM database (32). These databases provide predictions based on structural information from the Protein Data Bank (33). Human and parasites protein domains were predicted using Pfam scan, which combines the HMMER tool (34), and the domain models from Pfam version 31 (35). Linear motif–domain interactions are predicted using the regular expressions provided in the ELM database. We decided that a protein-protein interaction is supported by domain-domain and/or linear motif-domain interactions whether the interacting domains or linear motif-domain interactions reported by the databases (iPfam, 3did, ELM) appeared in the predicted host-parasite interaction. These data are available in the web resource: https://orthohpi.jensenlab.org/ the tab separated files (tsv) downloadable in the web contain a column (#11) indicating which of the predicted interactions are supported by interacting domains or domain-motif pairs.

### Network Analysis

Once we obtained the predicted host–parasite PPI networks, we used the topology of the network to identify relevant proteins that may play critical roles in the host–parasite crosstalk. There are several centrality measures that can be used to reveal node importance based on different node attributes such as degree. These different measures correlate to some extent and may highlight other nodes (36). Here, we used betweenness centrality to pinpoint proteins whose targeting would most disrupt this communication (37) but provide the code to generate several correlation measures (Supplementary File 1) with the provided networks in OrthoHPI website (http://orthohpi.jensenlab.org).

To identify key biological processes enriched in the predicted host–parasite PPI networks, we performed functional enrichment analysis using Gene Ontology terms (biological processes) (38) and Reactome pathway annotations (39). The lack of existing annotations in the parasite species limited the analysis to only the human proteins predicted to interact. This analysis revealed common and specific biological processes and pathways targeted by parasites. The functional enrichment was performed using Fisher's exact test and correction for multiple testing (Benjamini–Hochberg; FDR < 0.05). The enrichment was calculated using as background only the filtered proteome.

To overcome the lack of functional characterization of the studied parasites, we also investigated the functional classification of COGs (40) provided by the eggNOG database (29). These annotations classify COGs into broad functional categories that can be used to characterize the proteins grouped in these clusters (Supplementary Figure 1). These categories were transferred to the parasite proteins in the network and when the category was “Function unknown” we assigned as putative functions the categories of their interaction partners in human (Supplementary Table 1).

### Web Interface

To provide access to the predictions generated by our approach, we developed a web interface for OrthoHPI (http://orthohpi.jensenlab.org/). This web site provides interactive, predicted host–parasite PPI networks, which are visualized with d3.js (https://d3js.org/) and allow the user to easily navigate the full networks as well as the tissue-specific ones. The nodes in these networks represent parasite and human proteins and their sizes correspond to their betweenness centrality in the network. The edges between nodes show predicted molecular host–parasite interactions and are weighted using the maximum score transferred from STRING database. The predictions can be downloaded in tab-separated values file format or in Graph Modeling Language format, which is compatible with Cytoscape (41).

## Results

### Computational Prediction of 15 Host–Parasite Interactomes

We applied our integrative orthology-based approach to predict the host–parasite PPIs networks for 18 different eukaryotic parasites and obtained predicted PPIs for 15 of them (Table 1 and Figure 1). PPIs for *Entamoeba histolytica, Trichomonas vaginalis*, and *Leishmania major* could not be predicted due to the lack of orthologs. Our predictions returned a total of 27,352 interactions for 14,340 proteins (12,218 host proteins, 2,122 parasite proteins) being *T. gondii* and *C. hominis* the largest and the smallest predicted PPIs networks, respectively (Table 2). The large differences in the number of proteins in parasites and host after filtering process depend on whether the parasite is unicellular (filtering process included membrane and secreted proteins) or multicellular (filtering process included only secreted proteins) and in the number of human tissues related with the parasites tropism (Table 2). Our prediction approach transferred most of the STRING high-confidence intra-species interactions from model organisms (*M. musculus, S. cerevisiae, D. melanogaster, D. discoideum*, etc.) and *Homo sapiens* (Supplementary Figure 2).

**Table 1:** Human parasites analyzed.

**Tax_id**	**Species**	**E/I**	**U/M**	**Tissues**	**Proteome size**	**SS (%)**	**MP (%)**	**Input**
5691	*Trypanosoma brucei*	E	U	Skin, blood, brain, lymph node, spinal cord	8,747	708 (8, 1)	1,439 (16, 4)	SS+MP
353153	*Trypanosoma cruzi*	I	U	Skin, heart, blood, eye,	19,602	2,633 (13, 4)	3,373 (17, 2)	SS+MP
6334	*Trichinella spiralis*	E	M	Intestine, brain, heart, muscle, lung	16,380	705 (4, 3)	N/A	SS
6183	*Schistosoma mansoni*	E	M	Skin, liver, intestine, lung, blood	11,770	318 (2, 7)	N/A	SS
184922	*Giardia lamblia*	E	U	Intestine	6,502	407 (6, 2)	727 (11, 2)	SS+MP
5833	*Plasmodium falciparum*	I	U	Skin, blood, liver	5,429	619 (11, 4)	1,355 (25, 0)	SS+MP
5855	*Plasmodium vivax*	I	U	Skin, blood, liver	5,050	553 (11, 0)	900 (17, 8)	SS+MP
5850	*Plasmodium knowlesi*	I	U	Skin, blood, liver	5,102	565 (11, 0)	890 (17, 4)	SS+MP
353151	*Cryptosporidium hominis*	E	U	Intestine	3,885	324 (8, 3)	641 (16, 5)	SS+MP
353152	*Cryptosporidum parvum*	E	U	Intestine	3,805	397 (10, 4)	678 (17, 8)	SS+MP
5811	*Toxoplasma gondii*	I	U	Blood, brain, eye, heart, muscle, placenta	7,988	658 (8, 2)	1,188 (14, 8)	SS+MP
420245	*Leishmania braziliensis*	I	U	Skin, nose, mouth, blood	8,160	294 (3, 6)	1,152 (14, 1)	SS+MP
929439	*Leishmania mexicana*	I	U	Skin, nose, mouth, blood	8,147	305 (3, 7)	1,186 (14, 5)	SS+MP
5661	*Leishmania donovani*	I	U	Skin, liver, spleen, blood, bone marrow	8,032	310 (3, 8)	1,104 (13, 7)	SS+MP
435258	*Leishmania infantum*	I	U	Skin, liver, spleen, blood, bone marrow	8,150	325 (3, 9)	1,159 (14, 2)	SS+MP

*E, Extracellular; I, intracellular. U, Unicellular; M, Multicellular. Tissues: different tissues associated with the parasite's tropism (information retrieved from: https://www.cdc.gov/parasites/). Proteome size: sequences in the predicted proteome. SS, Soluble secretome; MP, Membrane proteins (%: is the percentage in relation to the overall proteome); N/A, Not applicable*.

**Table 2:** Human–parasite PPI networks.

**Species**	**Parasite proteins**	**Host proteins**	**Parasite nodes**	**Host nodes**	**Total nodes**	**Total edges**	**Avg. degree**
*T. brucei*	2,147	3,568	237	555	792	1,241	1.56
*T. cruzi*	6,006	5,046	123	954	1,077	2,333	2.16
*T. spiralis*	705	3,715	131	801	932	1,496	1.60
*S. mansoni*	318	3,175	74	491	565	695	1.23
*G. lamblia*	1,134	554	10	8	18	21	1.16
*P. falciparum*	1,974	6,979	270	1,235	1,505	2,659	1.76
*P. vivax*	1,453	6,979	349	1,716	2,065	4,163	2.01
*P. knowlesi*	1,455	6,979	376	1,756	2,132	4,470	2.09
*C. hominis*	965	554	3	13	16	13	0.81
*C. parvum*	1,075	554	7	37	44	44	1.0
*T. gondii*	1,846	11,796	351	2,556	2,907	6,863	2.36
*L. braziliensis*	1,446	4,073	35	239	274	388	1.41
*L. infantum*	1,484	7,065	58	840	898	1,273	1.41
*L. donovani*	1,414	7,605	54	647	701	1,039	1.48
*L. mexicana*	1,491	4,073	44	370	414	654	1.57

*This table contains the number of host and parasite nodes, the number of interactions, and the average degree of the predicted interactomes*.

The resulting host–parasite PPIs networks were analyzed to identify central proteins in the networks, tissue-specific connections, and enriched biological processes and pathways. The lack of experimentally validated host–parasite interactions that could be used as a gold standard prevented direct validation of the quality of the predicted interactions. Instead, we evaluated the plausibility of the network by looking at the known functions in which the involved proteins participate. The analyses are divided into two: a global study of the common mechanisms targeted by the studied parasites and the shared human interactors. We also provide a study case focusing on a closer examination of the human–*S. mansoni* predicted interactome.

### Common and Specific Mechanisms Targeted by the Studied Parasites

**Figure 2:**
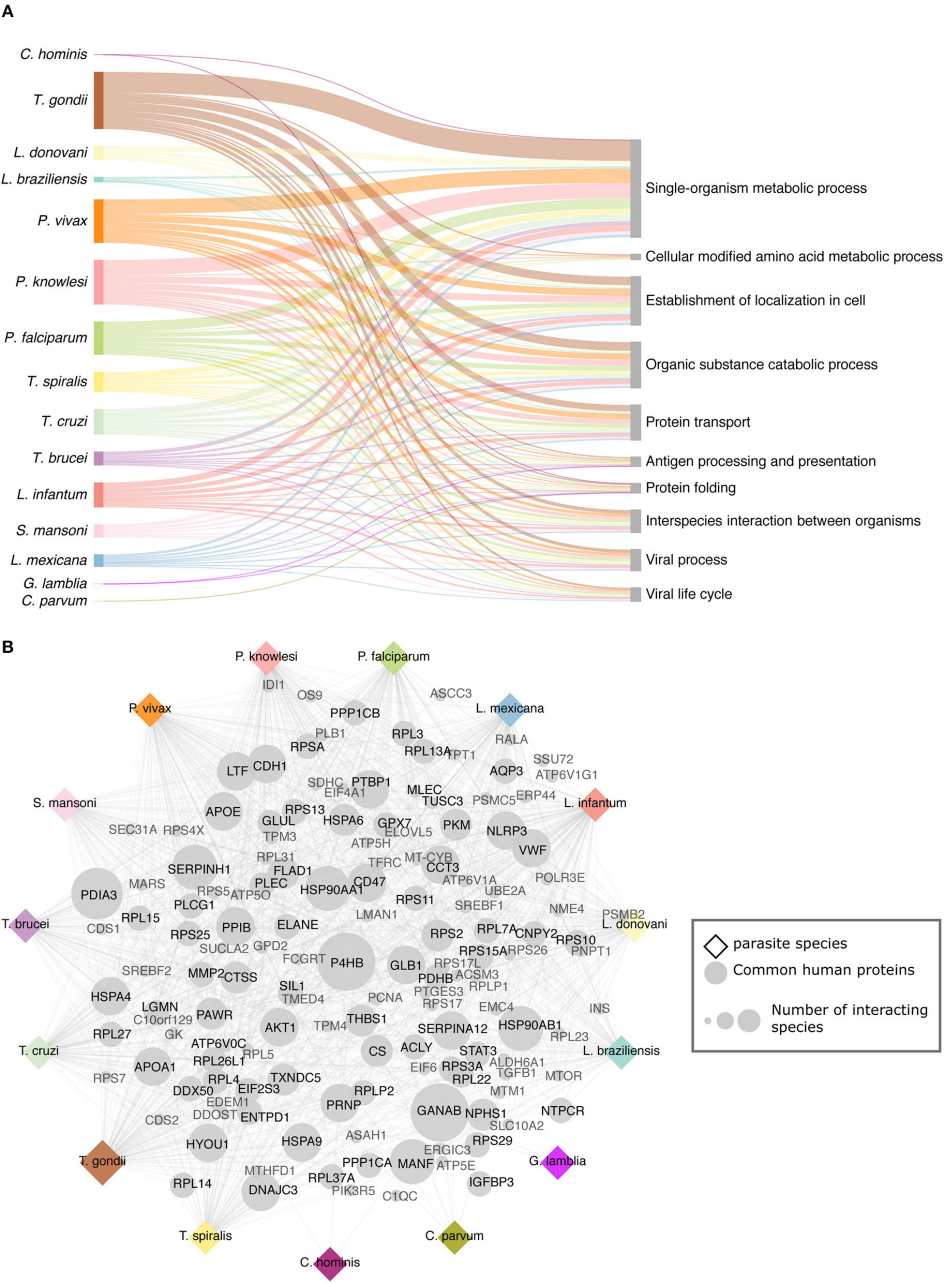
Targeted host processes and proteins. **(A)** Common and most relevant human biological processes (GO BP) targeted by the parasites. **(B)** Common human proteins across interactomes some of which have been already linked to pathogenic activity. Both the targeted processes and pathways and these common host interacting proteins may open new therapeutic possibilities.

We used annotations from both biological processes GO terms and Reactome pathways in human to get an overview of the shared pathways targeted by the studied parasites (Figure 2A). In total, 1,910 GO terms (biological process) were identified in human proteins targeted by parasites across all the interactomes (Supplementary Table 2). In the analysis, we found the biological process *Protein folding* enriched across 14 interactomes (Figure 2A). This biological process has been identified already as enriched and crucial in several host–pathogen systems, including mouse–*P. falciparum* (18); human–*M. tuberculosis* (42); *B. glabrata*–*S. mansoni* (43); *L. salmonis*–*F. margolisi* (44), based on transcriptomics and proteomics data. The protein folding biological process appears to be a conserved natural response to the infection and is related to a response to stress.

Different lifestyles (intra- and extracellular parasites) may define specific targeted pathways in the host. We analyzed the common processes and pathways enriched in intracellular and extracellular parasites specifically. The human GO terms and pathways enrichment analysis on networks for the extracellular parasites did not suggest any lifestyle-specific targeted mechanisms. However, the specific lifestyle requirements of the intracellular parasites revealed two (*thioester and acyl-CoA metabolic process*) exclusive biological processes related to exploitative mechanisms to acquire nutrients from the cytosol of host cells (i.e., *de novo* lipid synthesis) carrying out a type of sustainability interactions. In terms of host immune response to parasitic infection we obtained a Reactome pathway common to intracellular parasites: *antigen presentation: folding, assembly, and peptide loading of class I MHC*. This pathway belongs to the human innate immune defense by acting as pattern-recognition receptors, in particular against intracellular pathogens.

The host–parasite interactome of the intracellular *P. falciparum* has been to some extent studied both experimentally and computationally (13, 17, 23, 45–47). In order to establish a successful infection, *P. falciparum* proteins interact with a variety of human proteins on the surface of different cell types, as well as with proteins inside the host cells (47). For instance, the parasitophorous vacuole protein called ETRAMP5 was found to interact with the human apoliproteins ApoA, ApoB, and ApoE, which may be involved in the invasion of liver cells by sporozoites (47). Alternatively, this interaction may be important for parasite survival inside red blood cells or hepatocytes (47). Similarly, our results predict the interaction of human APOB with *P. falciparum* endoplasmin (PFL1070C) and phospholipase (PFF1420w) both parasitophorous vacuole proteins. These interactions were also predicted in liver tissue, which may be related to the parasitophorous vacuole function.

Cell signaling and cell adhesion have also been identified as a relevant biological processes targeted in the interaction between human and *P. falciparum* proteins. For instance, the parasite chaperone PFI0875w was found to interact with many prominent regulation and signaling host proteins such as members of the TNF pathway (13). Our results predicted the interaction between PFI0875w and human HSP70 protein, which promotes TNF-mediated apoptosis. Similarly, cell adhesion is crucial for host cell invasion and has been shown previously in receptor-mediated viral infections. For example, human protein CD55 (complement decay-accelerating factor) was related to infection by coxsackievirus (23). In *P. falciparum* protein TRAP (PFC0640w) was found to interact with leucine-rich proteins involved in cell adhesion (13). In our study, the same protein TRAP and a reticulocyte binding protein (PFD0110w) were predicted to interact with receptor proteins involved as well in viral infection like integrins and may play a role in the initial process of cell invasion by *P. falciparum* (48).

Across the *Leishmania* species, we identified a genus-specific biological processes related to lipid and fatty acid metabolism. In species that cause mucocutaneous leishmaniasis (*L. braziliensis, L. mexicana*), we found nine human specific processes, interestingly many of them related to cellular oxygen levels such as: *cellular response to decreased oxygen levels, cellular response to oxygen levels, negative regulation of cellular respiration*, and *cellular response to hypoxia*. Regarding visceral leishmaniasis (*L. donovani, L. infantum*) we found 40 specific GO terms associated with a relevant biological process in the host–parasite interaction such as *regulation of defense response to virus by virus* and *receptor-mediated endocytosis*.

In helminths (*S. mansoni*, and *T. spiralis*) we identified 24 specific biological processes, some of which were involved in specific process related to blood tissue, for example, *platelet degranulation* and *blood vessel development*. Several helminth parasites imbibe host blood, including hookworms, the flukes, and the major livestock nematode parasites (50). Helminth migration through different organs requires the degradation of the extracellular matrix and the disruption of cell junctions by some secreted proteolytic enzymes, causing damage along the path of the migration (51). According to our results, we found some GO terms enriched probably related to helminth migration such as *extracellular matrix disassembly* and *regulation of cell adhesion* to be enriched.

We studied the common host proteins to all or most of the studied parasites (Figure 2B). In all the inferred interactomes, GANAB (neutral alpha glucosidase AB) and P4HB (protein disulfide isomerase) proteins were predicted to interact with parasite proteins. GANAB protein is related to the host defense mechanisms and P4HB is relevant in the internalization of broad spectrum of pathogens such as *Leishmania*, HIV, dengue virus, and rotavirus.

### The Human–*S. mansoni* Interactome Reveals Relevant Central Proteins

**Figure 3:**
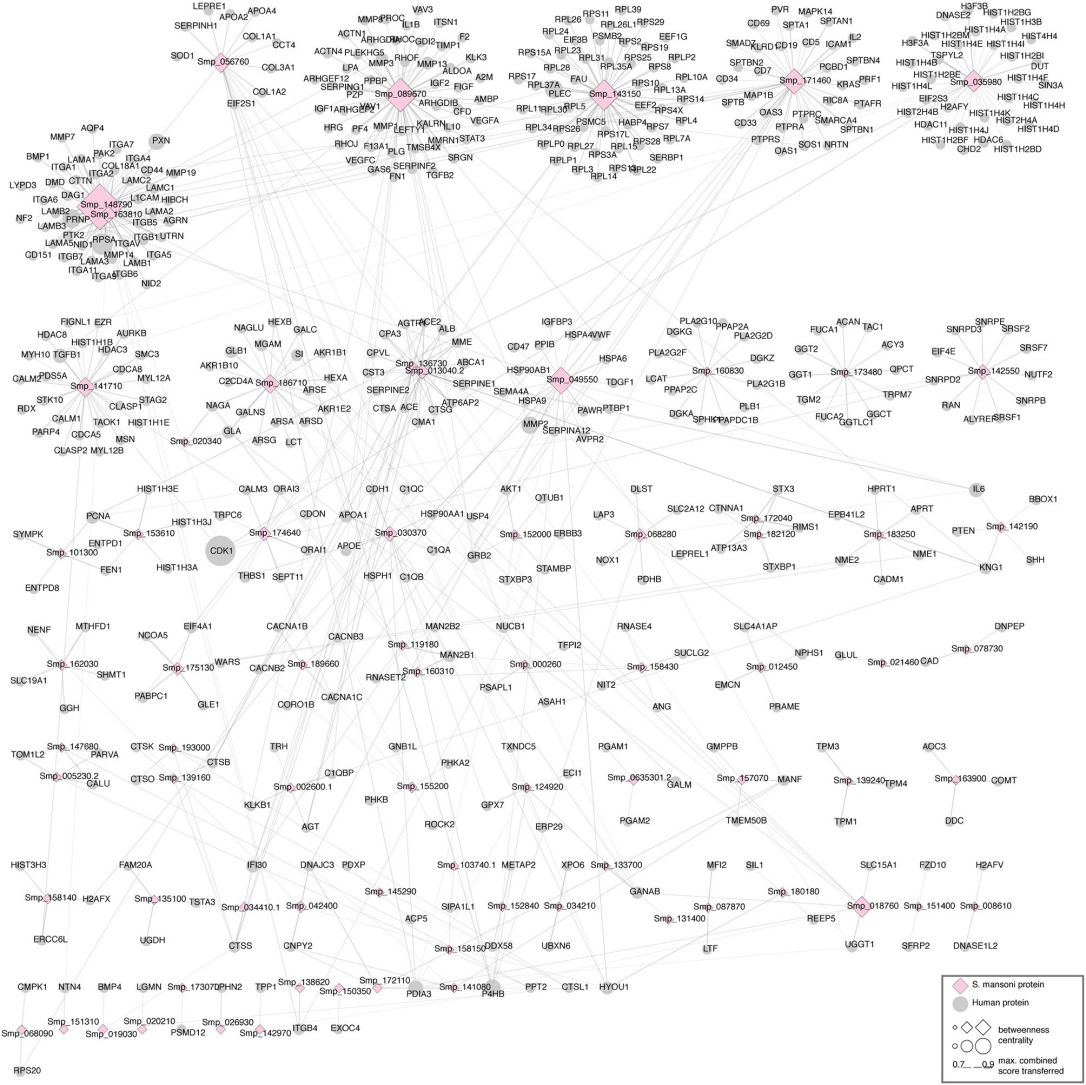
Human–*S. mansoni* predicted interactome. The layout of the nodes in the network (ellipses: human proteins, diamonds: *S. mansoni* proteins) uses Markov Clustering to group and locate the nodes. The tools used to visualize the networks in this article are: Cytoscape (41) and the clusterMaker app (49). The central nodes are calculated using betweenness centrality and highlighted in the figure with a higher size.

We identified 695 interactions (491 host proteins, 74 *S. mansoni* proteins), and 178 were supported by domain-domain and domain-linear motif interactions (https://orthohpi.jensenlab.org/). In this analysis, we use the topological characteristics of the predicted human–*S. mansoni* PPI network to identify central proteins. Network centrality helped to prioritize proteins by identifying nodes with a relevant role in the communication flow in the network, which may translate into biological relevant essentiality (52, 53). In the human–*S. mansoni* interactome network (Figure 3) the nodes with the highest centrality are shown in Table 3 and Figure 3. Within the Gene Ontology biological process, we found 325 gene ontology terms enriched in *S. mansoni* targets in the host (Supplementary Table 2). GO terms associated with human proteins involved with host–parasite interaction were also retrieved (Table 4). PPIs for each term are available in Supplementary Table 3.

**Table 3:** Top 10 of proteins with the highest centrality in the human–*S. mansoni* interactome.

**Protein ID**	**Description**
**Human proteins with the highest centrality**	
ENSP00000346067	Ribosomal protein SA
ENSP00000378699	Cyclin-dependent kinase 1
***S. mansoni*** **proteins with the highest centrality**	
Smp_056760	Protein disulfide-isomerase
Smp_018760	Neutral alpha-glucosidase ab
Smp_035980	Histone H2A
Smp_171460	Cell adhesion molecule
Smp_049550	Heat shock protein 70 (Hsp70)
Smp_143150	Eukaryotic translation elongation factor
Smp_089670	Alpha-2 macroglobulin
Smp_148790	Laminin subunit beta 1

**Table 4:** GO term enrichment analysis of the most relevant biological process in the *H. sapiens*—*S. mansoni* interactome.

**GO term**	**Description**
GO:0002251	Organ or tissue specific immune response
GO:0002227	Innate immune response in mucosa
GO:0030198	Extracellular matrix organization
GO:0022617	Extracellular matrix disassembly
GO:0007160	Cell-matrix adhesion
GO:0031589	Cell-substrate adhesion
GO:0044419	Interspecies interaction between organisms
GO:0016032	Viral process
GO:0033554	Cellular response to stress
GO:0071496	Cellular response to external stimulus
GO:0007596	Blood coagulation
GO:0003073	Regulation of systemic arterial blood pressure
GO:0007229	Integrin-mediated signaling pathway
GO:0009611	Response to wounding

*PPIs for each term are available (Supplementary Table 3)*.

The human–*S. mansoni* interaction network is not static and the tissue expression data used to filter the host proteome can also be used to give context to the predicted molecular associations. Modeling spatial context into the predicted network allowed the identification of relevant interactions associated to tissues through the parasite's life cycle. 1,096 interactions were identified in five tissues related with the parasite tropism (Intestine 114, lung 191, blood 195, liver 443, skin 153) (see https://orthohpi.jensenlab.org/) (Table 5). Top 10 of highest centrality proteins were also identified in every tissue network (Table 5) and two central proteins were conserved across five tissues: Smp_089670 (Alpha-2 macroglobulin) and Smp_171460 (Cell adhesion molecule).

**Table 5:** Human–*S. mansoni* tissues PPI networks.

**Tissues**	***S. mansoni* nodes**	***H. sapiens* nodes**	**Total nodes**	**Total edges**	**Avg. degree**	**Top 10 (HC Proteins)**
Blood	41	131	172	195	1.13	Smp_030370 Smp_049550 Smp_089670 Smp_141710 Smp_148790 Smp_171460 ENSP00000221930 ENSP00000295897 ENSP00000339007 ENSP00000368752
Intestine	38	77	115	114	0.99	Smp_049550 Smp_089670 Smp_018760 Smp_141710 Smp_143150 Smp_171460 Smp_186710 ENSP00000221930 ENSP00000327801 ENSP00000368678
Liver	68	305	373	443	1.18	Smp_018760 Smp_030370 Smp_056760 Smp_089670 Smp_143150 Smp_148790 Smp_163810 Smp_171460 ENSP00000228307 ENSP00000346067
Lung	49	127	176	191	1.08	Smp_049550 Smp_089670 Smp_141710 Smp_148790 Smp_171460 Smp_174640 ENSP00000261405 ENSP00000266376 ENSP00000291295 ENSP00000339007
Skin	49	103	152	153	1.00	Smp_049550 Smp_089670 Smp_141710 Smp_148790 Smp_171460 ENSP00000339007 ENSP00000341189 ENSP00000378699 ENSP00000384886 ENSP00000398632

*Human and S. mansoni identifiers were retrieved from TISSUES and STRING databases, respectively. HC, Highest centrality*.

**Figure 4:**
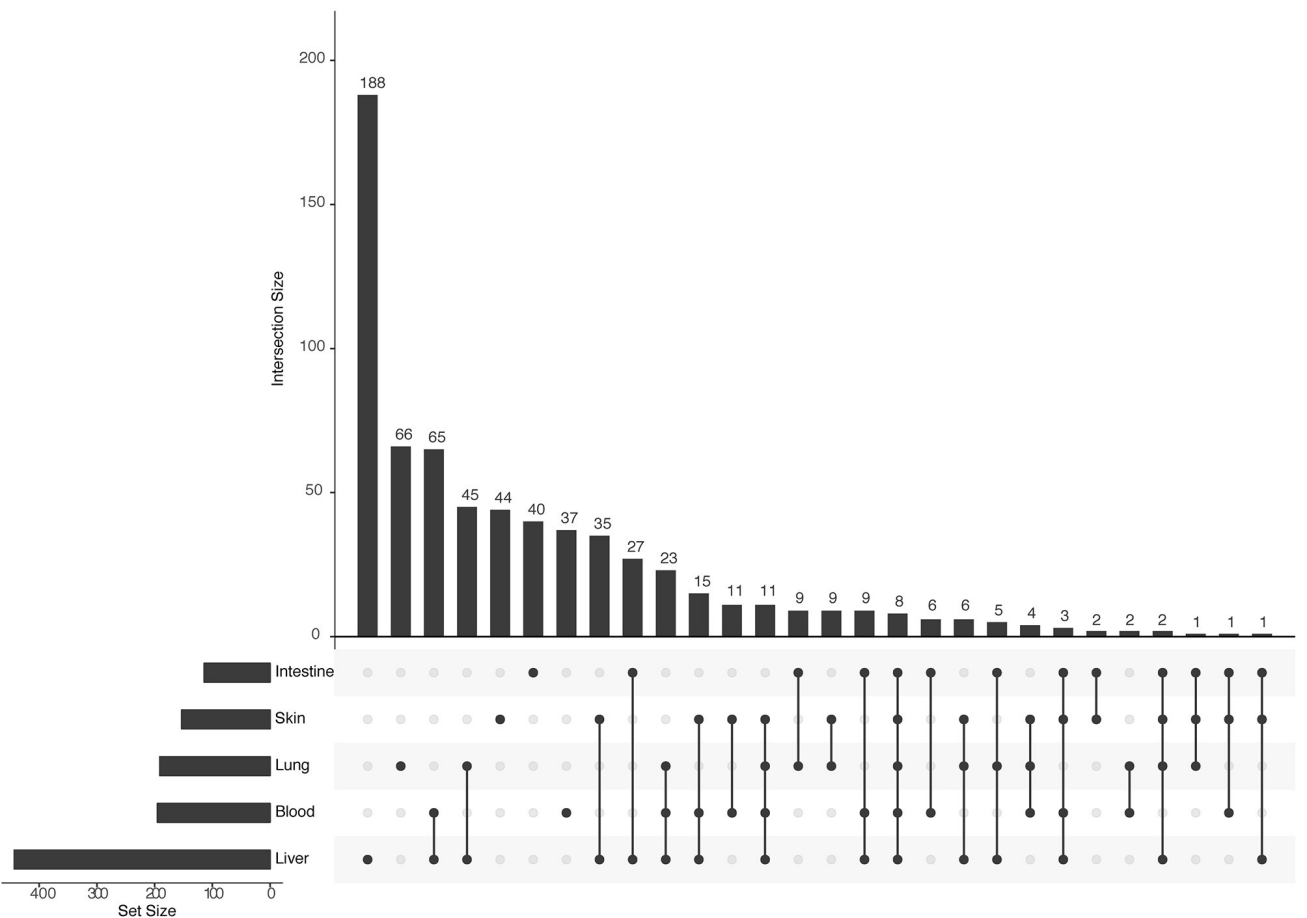
Upset plot of tissue-specific interactions in the human–*S. mansoni* predicted interactome. The bar chart on the left indicates the total of interactions in each of the five tissues associated with *S. mansoni* tropism: Liver is the tissue with the most interactions (443 interactions). The upper bar chart indicates the intersection size of shared interactions across tissues and tissue-specific interactions. Blood and Liver are the tissues with more shared interactions (65 interactions) and liver is the tissue with the most tissue-specific interactions (188 interactions).

Tissue-specific interactions are relevant to know the specificities in different relevant tissues related to *S. mansoni* lifecycle. In total, we found 375 tissue-specific interactions (skin 44, lung 66, liver 188, intestine 40, blood 37) (Figure 4) (Supplementary Table 4). Eight interactions were conserved across tissues (blood, intestine, liver, lung, skin) (Supplementary Table 4). Four *S. mansoni* proteins (Smp_089670, Smp_018760, Smp_049550, Smp_056760), which were part of the eight conserved interactions across tissues are central proteins in the whole interactome human–*S. mansoni*. P4HB a common targeted host protein across the 15 interactomes appeared in six of the eight interactions conserved across five tissues.

#### The OrthoHPI Web Resource

**Figure 5:**
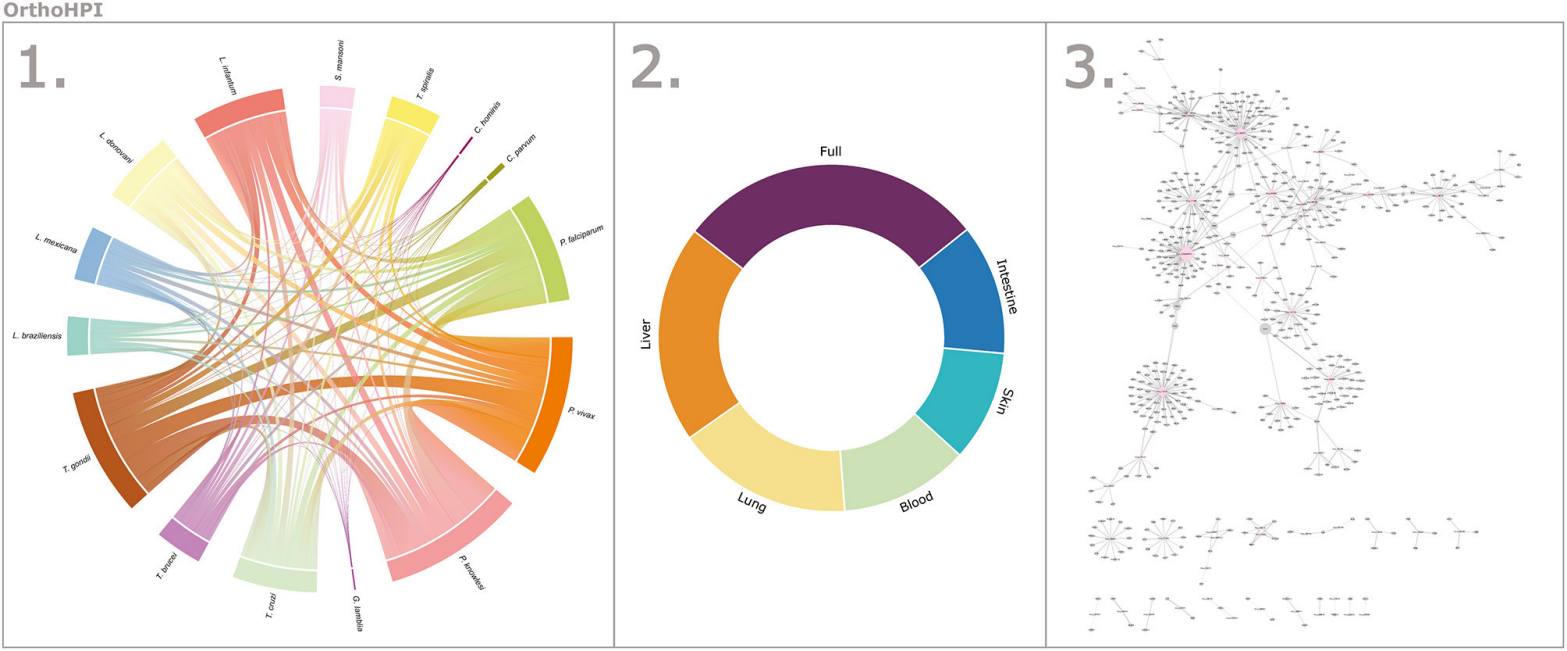
The OrthoHPI web resource. The website (http://orthohpi.jensenlab.org) allows you to visualize the predicted interactomes for all the studied parasites in three simple steps. **(1)** Select parasite, **(2)** Choose to visualize the full network or a tissue–associated interactions, and **(3)** Visualize the interactive network and download the interactions.

We developed a web resource (http://orthohpi.jensenlab.org) to provide all the predicted interactomes for all the studied parasites (Figure 5). The aim of OrthoHPI is to facilitate the analysis of the predicted host–parasite PPI networks, provide easy access to the full and tissue-specific networks and produce visual, navigable, and interactive networks easily manipulated by users.

## Discussion

The efficacy of treatments for parasitic diseases is still limited, and in many cases, parasites develop resistance. Thus, there is an urgent need to discover novel drugs or vaccines for these neglected diseases. However, our understanding of host–parasite molecular crosstalk is still very limited, as only a few systematic experimental studies have so far been performed. Being this the main reason to predict PPIs using computational approaches.

Here, we predicted host–parasite PPI networks for 15 diverse parasitic species and studied the functional relevance of these interactions by analyzing enriched processes among the human proteins predicted to interact with parasite proteins. The different enrichment analyses allowed us to compare and identify commonalities and specificities across the studied parasites. Additionally, these analyses revealed biological processes and metabolic pathways previously described in the literature as being affected during parasitic infection, which helped to validate functionally the predicted interactions. In the case of the most studied parasite from our list, namely *P. falciparum*, we were able to compare the list of GO terms enriched in the human-*P. falciparum* predicted PPI network with an already published functional study (13). In this study, the authors report a list of GO biological processes in human that are most affected by the interaction with *P. falciparum* (100 GO terms). From this list, 51 terms were in common with our results. The overlapping terms were related mainly with intracellular process in general such as to intracellular transport, signal transduction, cell cycle, actin filament-based process among others. The remaining non-overlapping 49 terms were related with more specific biological process targeted by *P. falciparum* such as cell death, apoptosis, intracellular protein kinase cascade among other (Supplementary Table 5).

The 15 parasites included in our study are different regarding phylogeny and biology, for example, distinct forms of invasion and the invasion of different types of host tissues (Table 1). Nonetheless, when combining all the predicted PPI networks, we were able to identify common core pathways and biological processes, which were targeted in the host by all the studied parasites. Indeed, it has been previously shown that evolutionarily distinct parasites can target the same pathways as a result of convergent evolution, for example in *Arabidopsis* pathogens, such as the bacterium *Pseudomonas syringae* and the eukaryote *Hyaloperonospora arabidopsidis* (54).

Eukaryotic pathogens are able to synthesize a number of nutrients required for *de novo* growth*;* however, it is more advantageous for them to conserve energy and take host derived resources (55, 56). The enriched processes in intracellular parasites are related to the acquisition of host lipids necessary for the parasites to assemble a large amount of new membranes during replication within host cells. The uptake of host lipids may be associated with how *Leishmania* species deplete membrane cholesterol and disrupt lipid rafts in host macrophages during the invasion process (57).

Concerning the host immune response to parasitic infection we found one Reactome pathway that is common to intracellular parasites: antigen presentation, folding, assembly, and peptide loading of class I MHC. This suggests that innate sensing of parasites is important for the induction of pro-inflammatory responses aimed at controlling infection (56, 58) and belong to the innate immune defense by acting as pattern-recognition receptors, in particular against intracellular pathogens (59) and parasites as the analyzed in this work (60).

In mucocutaneous leishmaniasis, the enriched process make sense, since in skin infected wounds, low oxygen tensions (hypoxic conditions) prevail, whereas oxygen supply promotes wound healing, and helps to control infections (61). Hypoxia can have multiple effects on host–pathogen interactions, many intracellular pathogens successfully adapt to hypoxic conditions and according to our results, the enriched process are showing a kind of response from the host based on the proteins that are part of these enriched biological process.

For intracellular parasites, the first challenge during infection is to gain access to the intracellular environment. Cell invasion starts when parasites contact the surface of the host cell and cellular receptors mediate parasite internalization (62). Consistent with this, we found the GO term *receptor-mediated endocytosis* to be significantly enriched in the PPI networks for *Leishmania* parasites.

Some parasites, such as schistosomes, are in constant contact with blood as they inhabit the host's veins. Blood coagulation is triggered by several pathways, which are targeted by blood-feeding parasites to inhibit coagulation and prolong blood flow (50). In helminths, we found specific biological process (*blood vessel development* and *platelet degranulation)* related to the inhibition of blood coagulation, which is also related to proteinases that facilitate the invasion of host tissues and digest host proteins. Additionally, parasite proteinases help pathogens evade the host immune response and prevent blood coagulation (63). Helminths-specific enriched Reactome pathways targeted two host mechanisms, sphingolipid metabolism, and glycosphingolipid metabolism, which are known to have a key role in the interaction host–helminths.

In addition to metabolic pathways and GO terms shared by parasites, certain host proteins targeted by parasites were also recurring across interactomes. One of them is protein disulfide isomerase (P4HB), which plays a key role in the internalization of certain pathogens (64). For example, during the host invasion process of *L. chagasi* increased levels of P4HB were found to induced phagocytosis in the promastigote phase and inhibition of expression of this gene reduced phagocytosis (65). The role of P4HB has also been associated with other pathogens such as HIV, dengue virus, or rotavirus (66–68). In dengue virus, P4HB was linked to a reduction of β1 and β3 integrins allowing for the entry of the virus (68) and in MA104 cells, thiol blockers, and P4HB inhibitors decreased the entry of rotavirus (66). The other recurring human protein is GANAB, which is related to the alteration of eosinophil proteome (69). Strikingly, eosinophils are important mediators of allergies, asthma, and adverse drug reactions, and are also related to the host defense mechanisms against helminth infections, which are characterized by eosinophilia (70–72). Straub et al. (69) conducted a comparative proteomic analysis of eosinophils (healthy vs. hypereosinophilia from acute fascioliasis), and GANAB was one of the four proteins significantly upregulated in the *Fasciola*-infected patient.

These results show that GANAB and P4HB are relevant proteins not only in our 15 interactomes but also in other host-pathogen systems, which confirm these proteins as possible hallmarks of the host–parasite interaction. Other host proteins that were not recurring across the 15 interactomes but common in other interactomes (Figure 2B) such as HYOU1, PDIA3, HSPA9, CTSS, etc., have been already also found deregulated upon pathogenic infection (73–78).

According to our network topology results, we chose betweenness centrality as a measure to predict central proteins in the human-*S. mansoni* interactome, there are multiple network centrality measures, and they do correlate to some degree (79). We have generated a correlation analysis between different centrality measures for human-*S. mansoni* interactome (Supplementary Figure 3). In this case, we chose betweenness centrality because we are interested in highlighting nodes that are central to information flow in the network, which may translate into more relevant nodes for the human-*S. mansoni* interactome. We identified central proteins in the human–*S. mansoni* PPI network (Table 3) involved in crucial biological processes needed for invasion and survival of the parasite: protease activity regulation, inhibition of blood coagulation, cell adhesion, and migration (80).

Our results predict that the *S. mansoni* protein Alpha-2 macroglobulin (Smp_089670) interacts with host proteins involved in extracellular matrix organization such as SERPINE1 or metalloproteases (MMP2, MMP3, MMP13, MMP8, and MMP1). Interactions with the extracellular matrix (ECM) components influence several biological processes and ultimately the fate of the host cell (81). Parasites encounter the ECM as a barrier and they deploy several mechanisms to overcome it such as cell adhesion, induction of ECM degradation, and regulation of the immune response (4, 82, 83). For example, Alpha-2 macroglobulin inhibits the predicted five metalloproteases by a trapping mechanism that limits their access to substrates (80). Laminin subunit beta 1 (Smp_148790) interacts with host protein PLEC, which strengthens cells and tissues by acting as a cross-linking element of the cytoskeleton (84). This interaction may compromise cell and tissue integrity (cell junction) and may be used by the parasite to migrate across different host tissues. The alteration of cell junction and tissue remodeling has been observed in some helminth parasite infections (85). The cell adhesion protein (Smp_171460) is also involved in migration and has interacting partners belonging to the metalloprotease, collagenase, and laminin families, according our results. Histone H2A, another central protein has been identified as part of the secretome in *S. mansoni* mainly from eggs (86). This protein has moonlight functions outside of the nucleus in parasites and has evolved additional functions in invasion and interaction with the host (87).

Among the central proteins, we found several *S. mansoni* immunoreactive proteins, namely protein disulphide-isomerase, heat shock protein 70, and eukaryotic translation elongation factor. These proteins were experimentally supported as immunoreactive in a study using adult worm protein extracts probed with pooled sera of infected and non-infected (naturally resistant) individuals from an *S. mansoni* endemic area (88). Neutral alpha-glucosidase, a central protein in the *S. mansoni* network, was also detected as immunoreactive in *S. mekongii* in mouse and patient sera (89). These results showed that probably central proteins are more related to the stimulation of the immune response that non-central proteins in the host–*S*. *mansoni* interactome. Functional significance of a protein is related to its position in the PPI network, as deletion of hub proteins (central proteins) have more impact than non-hubs in the interactome, explaining the function essentiality of the central nodes (52). In our case probably are relevant proteins to keep the host–parasite interaction since pathogens may have adapted to “attack” proteins involved in specific pathways, most importantly in immunity and defense mechanisms (53) as evidenced in this work.

Regarding tissue-specific network analysis, we found that in the blood-specific PPI network, there are *H. sapiens* proteins targeted by *S. mansoni* involved in biological processes such as response to stress, blood coagulation, regulation of immune process among others (Supplementary Table 4). Two interactions between alpha 2 macroglobulin (Smp_089670) and coagulation factors like platelet factor four (ENSP00000296029) and Kallikrein (ENSP00000314151), could result in inhibition of the coagulation factors, which may facilitate the migration of the parasite through the broken tissue/vessels (80). Equally compelling is the predicted lungs-specific interaction between the host protein FZD10 (ENSP00000229030), previously implicated in the inflammatory response in the alveolus (90), and the parasite protein Wnt (Smp_151400).

Here, we identified relevant PPIs in the human–*S. mansoni* interactome related with different stages of the host–parasite interaction such as: adhesion, invasion, feeding, migration, immune evasion, and interactions with specific environments like tissue-specific interactions. Thus, the reported interactions can be used as a starting point in follow up experiments that could lead to better understanding of the disease pathogenesis and the parasite's biology, as well as open new frontiers in the identification of novel therapeutic targets against neglected diseases.

## Conclusion

In the present study, we provide 15 predicted host–parasite interactomes, a functional analysis of the main common and specific processes targeted by diverse parasites, and an in-depth analysis of the human–*S. mansoni* PPI network. As well, we highlighted biological processes, pathways, and tissue-specific interactions that may be essential in the life cycle of the parasites. Moreover, our results highlight mechanisms and specific components that may be candidates for the study of new druggable targets.

One of the advantages of our prediction method is that it combines an orthology method with biological context relevant in the parasite's life cycle, which provides higher quality by reducing noise. Further, we integrate high-quality information from well-known and benchmarked sources of orthology prediction, intra-species protein-protein interactions, and protein location. The approach is scalable, and can be applied to many different host–pathogen systems.

Our study could also be used to assign functions to parasite's hypothetical proteins, assuming that clustered proteins tend to have similar functions and functionally related proteins can interact with each other (91–93). This application would be useful in parasite genomics, considering that a large number of parasite proteins are annotated as hypothetical proteins, and our networks provides a useful resource for annotation of those proteins.

Despite the limitations of homology prediction methods that tend to yield a high number of false positives, our approach constrained the predictions by including parasite-specific biological context, which filtered out some of the interactions otherwise transferred by orthology but biologically inconsistent with the life cycle of the parasite. Unfortunately, the lack of experimental data limited the options to benchmark the accuracy of the method. However, we provide several evidence from scientific literature of known host-parasite protein-protein interactions and established biological processes targeted by the parasites, which we also predicted in this study. Hence, we believe that the predicted interactions provide a base for hypothesis generation and help to focus follow up experiments that can prove the interaction and also find new druggable targets (11). Additionally, the common and specific biological processes identified help to understand parasitic invasion, infection, and persistence in the host and thereby the biology of parasitic diseases.

## Author Contributions

YC-A, AS, GO, and LJ conceived and designed the experiments. YC-A performed the analysis of the biological relevance of the PPIs and contributed to the development of the pipeline. AS developed the pipeline and the web resource. All authors participated in reviewing and editing the manuscript and approved the final manuscript.

### Conflict of Interest Statement

The authors declare that the research was conducted in the absence of any commercial or financial relationships that could be construed as a potential conflict of interest.

## Supplementary Material

Supplementary File 1 Python script to analyze node centrality in the networks.

Supplementary Figure 1 Intraspecies eggNOG groups functional annotation transferred. Functions annotated in eggNOG to the most contributing clusters of orthologs groups (COGs) used in the orthology transfer method.

Supplementary Figure 2 Species that contributed the most to the PPI transfer. Most of the interologs transferred to the host–parasite system corresponds to model organisms, which account for most of the high confidence PPI in the STRING database.

Supplementary Figure 3 Correlation analysis between different centrality measures for human-*S. mansoni* interactome.

Supplementary Table 1 Annotation for parasites proteins with unknown function.

Supplementary Table 2 Human GO terms enriched targeted by 15 parasites. Biological process targeted by parasites across 15 interactomes. Numbers represent the proteins associated with each GO term.

Supplementary Table 3 PPIs of relevant human GO terms enriched in the human–*S. mansoni* interactome. These PPIs belong to enriched GO terms from Table 4.

Supplementary Table 4 Tissue-specific and shared interactions across five tissues in the human–*S. mansoni* interactome.

Supplementary Table 5 Comparison of human GO terms enriched targeted by *P. falciparum*. This comparison was made between results from Wuchty (13) and our results.
