# Supplementary figures and images for: Analysis of Predicted Host–Parasite Interactomes Reveals Commonalities and Specificities Related to Parasitic Lifestyle and Tissues Tropism
Source: Front Immunol. 2019 Feb 13;10:212. doi: 10.3389/fimmu.2019.00212 (PMC6381214; doi:10.3389/fimmu.2019.00212)

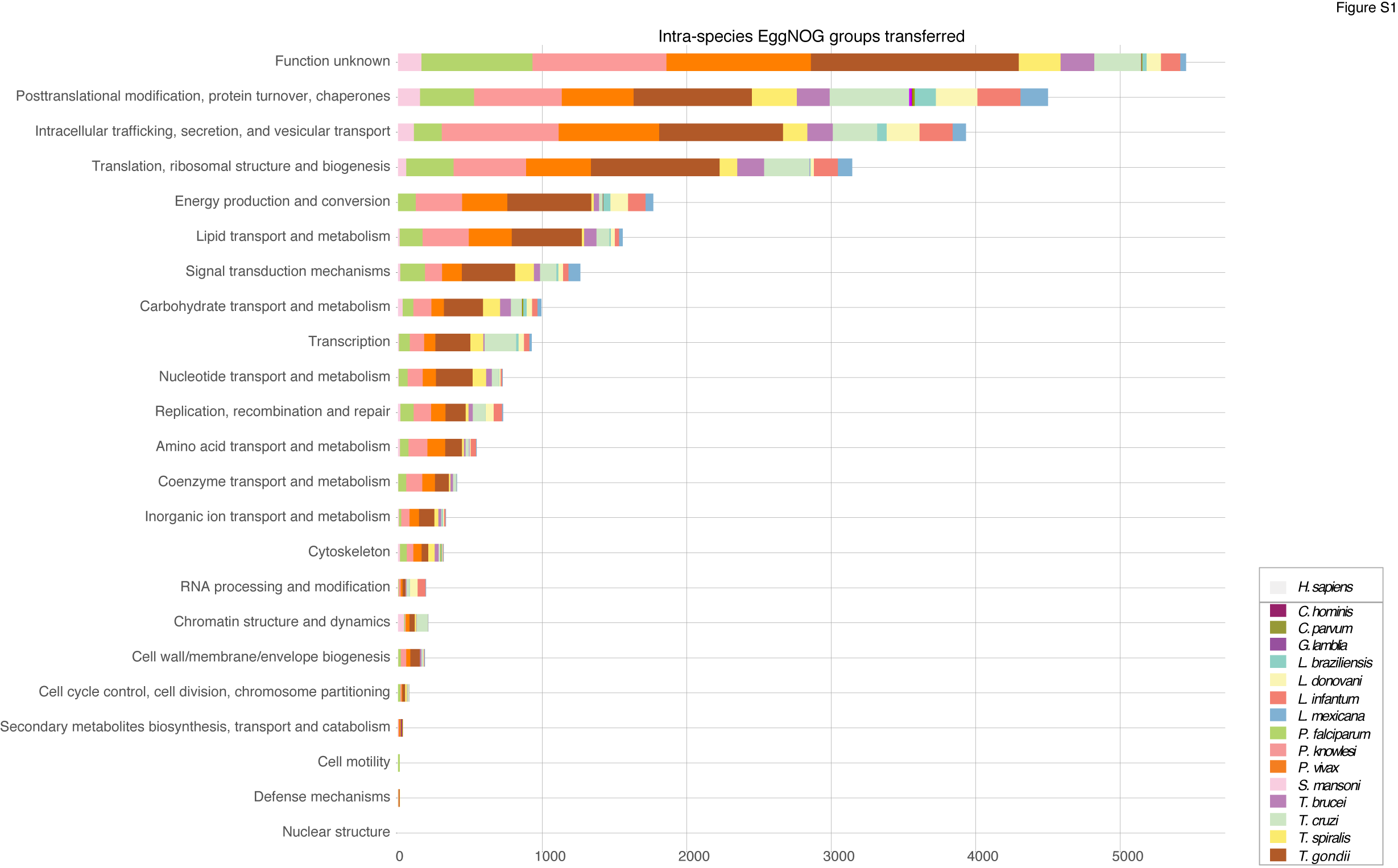

Supplement: Supplementary Figure 1 — Intraspecies eggNOG groups functional annotation transferred. Functions annotated in eggNOG to the most contributing clusters of orthologs groups (COGs) used in the orthology transfer method. [file Image_1.TIF]

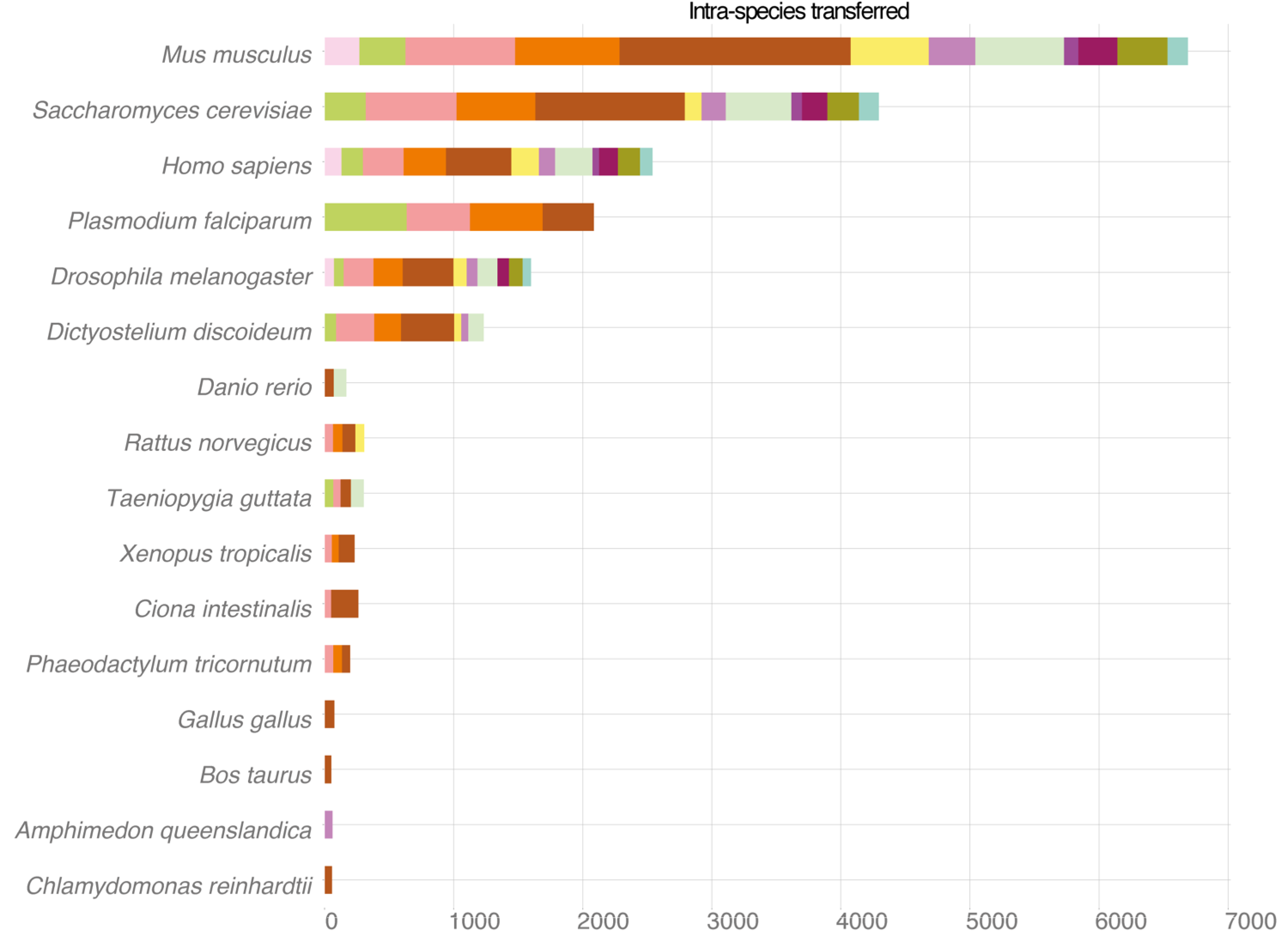

Supplement: Supplementary Figure 2 — Species that contributed the most to the PPI transfer. Most of the interologs transferred to the host–parasite system corresponds to model organisms, which account for most of the high confidence PPI in the STRING database. [file Image_2.TIF]

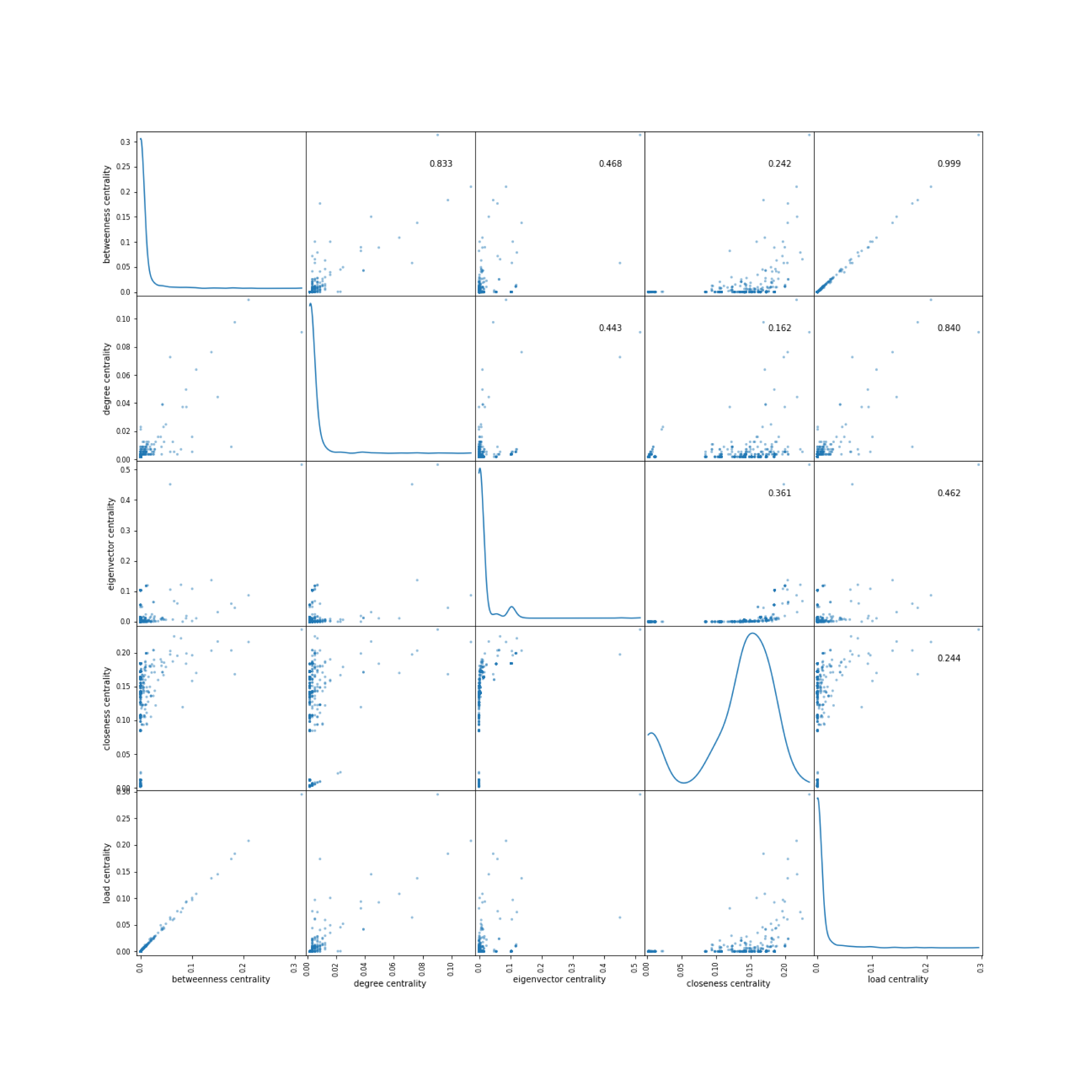

Supplement: Supplementary Figure 3 — Correlation analysis between different centrality measures for human-S. mansoni interactome. [file Image_3.TIF]
